# National study of child sexual abuse cases in Oman: Characteristics and medical-legal outcomes

**DOI:** 10.1016/j.heliyon.2024.e40434

**Published:** 2024-11-19

**Authors:** Muna Alshekaili, Mohammed Ali Al-Marzoqi, Salim Al-Huseini, M Mazharul Islam, Fatima Al-Sulaimani, Walid Hassan, Yahya Alkalbani, Mohamed Al Breiki, Abdullah Al-Madhani, Nithila Mariam Roy, Ibrahim Al-Zakwani, Aishwarya Ganesh, Samir Al-Adawi

**Affiliations:** a Al Masarra Hospital, Ministry of Health, Wilayat Al Amerat, Muscat, Oman; b Former Public Prosecutor, Oman's Judicial Authority, Madinat Sultan Qaboos Street, Muscat, 113, Oman; c Department of Statistics, College of Science, Sultan Qaboos University, Muscat, 123, Oman; d Ministry of Health, Madha, Musandam Governorate, Oman; e Ministry of Health, Sohar, North Batinah Government, Oman; f Department of Behavioral Medicine, College of Medicine & Health Sciences, Sultan Qaboos University, Al Khoudh, 123, Muscat, Oman; g Department of Pharmacology and Clinical Pharmacy, College of Medicine & Health Sciences, Sultan Qaboos University, Al Khoudh, 123, Muscat, Oman

**Keywords:** Child sexual abuse, Forensic examination, Legal outcome, Retrospective, Survey, Prosecution of assailants

## Abstract

**Background:**

To stay abreast of the best international practices, the Arab Gulf countries have ratified the United Nations *Convention on the Rights of the Child Treaty,* which includes clauses on safeguarding the well-being of children against child abuse and neglect. The enactment of laws, policies, and facilities designed to protect the rights of the child has not yet been studied to determine whether it leads to appropriate legal dispositions against perpetrators of child sexual abuse (CSA) in Oman.

**Aims:**

This study has been launched to address two interrelated objectives; (i) describe the characteristics of CSA victims and perpetrators and (ii) examine factors associated with medicolegal findings and judicial results for CSA complainants in Oman.

**Methods:**

Data come from a retrospective survey that covered one year from January 2017 to December 2017. Data from participants who met the study criteria were drawn from statistics published by the Oman Public Prosecution. The study was designed to obtain sociodemographic characteristics of victims and perpetrators of CSA, the types of prevalent cases of CSA, whether CSA was the result of family or extrafamilial encounters, and the characteristics of medical findings and legal results.

**Result:**

During the designated period, 269 victims and 269 defendants were identified. Most of the victims were boys (55.8 %), and their ages ranged from 9 to 15 years (mean age 12 years). Among the perpetrators, all were men, with ages ranging between 20 and 40 years (mean age 28.3 years). The vast majority (96.7 %) of reported cases were of extrafamilial type. The identified types of CSA constituted inappropriate sexual behavior towards children (63 %), followed by sodomization (26 %) and vaginal coitus (7.1 %). The factors that explained the results in favour of the victims in the medicolegal findings included the female sex of the victim (AOR = 2.78, 95%CI = 1.01–7.87), and the ages of the victim between 8 and 12 years (AOR = 4.29, 95 % CI = 1.51–12.16). Among the cases that were tried in court, 76 % were convicted and sentenced to an average of 30 months in correctional facilities. Factors associated with the conviction of the perpetrator were victims' ages between 13 and 17 years (AOR = 3.45, 95 % CI = 1.22 to 9.79), the type of abuse was sodomization (AOR = 1.95, 95%CI = 1.08–6.78), and cases that included positive forensic results (AOR = 2.66, 95 % CI = 1.23 to 8.45).

**Discussion:**

To date, studies on medical-legal and judicial outcomes of CSA in developing countries have received little attention. The present sentinel study lays the foundation for future studies using the robust methodology required to further scrutinize the medicolegal findings and judicial results of CSA in similar regions of the Middle East. The present findings also suggest that the CSA trends in Oman tend to echo those reported from developed countries, but there are also culture-specific factors that shape reporting, as well as medico-legal and judicial outcomes.

## 1 Introduction

The World Health Organization (WHO) has reported that 73 million boys and 150 million girls have been subjected to different forms of child sexual abuse (CSA) [1]. WHO defines sexual assault as cited by Krug, Mercy, Dahlberg & Zwi [2] as ‘*any sexual act, attempt to obtain a sexual act, unwanted sexual comments or advances, or act to traffic or otherwise directed against a person's sexuality using coercion, threats of harm or physical force, by any person regardless of their relationship with the victim, in any setting, including but not limited to home and work*’ (p.149). According to Simon, Luetzow & Conte [3], CSA tends to *'occur purely for the sexual gratification of the perpetrator*'. Arab Gulf countries are increasingly adopting best practices to protect the well-being of minors, including ratification of the ***Convention on the Rights of the Child*** (CRC) [4,5]. The CRC stipulates that authorities must take all appropriate legislative, administrative, social, and educational measures to protect the child from all forms of physical or mental violence, injury or abuse, neglect or negligent treatment, maltreatment, or exploitation, including sexual abuse (CRC Article 19) [6]. Several studies concluded that sexual assault during childhood triggers intransigent and debilitating physical and emotional sequelae [[7], [8], [9]]. This has the net effect of affecting social, occupational and educational competence. Physical sequelae can have various negative consequences, such as contracting sexually transmitted infections, chronic disability, and unwanted pregnancy [10,11]. Victims of CSA are also at increased risk of anxiety, depression, and suicidality. These consequences, in turn, are likely to increase healthcare utilization and negatively impact the quality of life and meaningful existence of victims [12].

Although a survey of key informants on child protection from various regions of the world appears to suggest that there is consensus on what constitutes sexual assault and that it is largely perceived as a criminal violation of human rights [13], sexual assaults around the world are likely to remain hidden from the medical services and the judicial system [14]. Mont & White [15] suggest that critical factors that contribute to the lack of prosecution of sexual assault cases around the world include the "three C" - (in)competence, contempt, and corruption – that often arise due to a lack of knowledge about the complexity of sexual assault and the tendency in patriarchal cultures to blame the victim of fraud in the medical and judicial systems. Saint-Martin, Bouyssy, & O'Byrne [16] have examined medicolegal findings and legal outcomes in France. These authors reported that 46.2 % of the cases did not lead to prosecution due to insufficient evidence and approximately 36 % of the assailants were convicted. Hagemann et al. [17] have surveyed the impact of forensic documents and the legal process in Sør-Trøndelag in Norway and, among identified police-reported cases, only 9.72 % of the cases led to prosecution and a guilty verdict. In Australia, Lewis, Klettke & Day [18] have examined the outcome of CSA and reported that the prosecution often relies on accrued behavioural data compared to biomedical evidence.

Although studies from Arab Gulf countries have suggested that the region is not immune to the vagaries of CSA [19], studies on the relevant legislative system for sexual assault are scarce. Existing studies, although small in number, suggest that even in highly industrialised regions of the global north, where well-developed judicial and police systems are in place, only a very small percentage of CSA cases achieve positive criminal justice outcomes. The present sentinel study aims to examine the characteristics of victims, perpetrators and medical-legal and judicial outcomes of CSA cases in Oman.

Oman is located on the southern coast of the Arabian Peninsula [20]. Islam is the dominant religion in Oman and is deeply embedded in its culture and legal system. The practice of Islam in Oman generally adheres to the Ibadi sect, along with a significant number of other sects [21]. Studies have shown that family and community bonds are highly valued in Omani society, with extended families being common [22]. This familial structure can influence the dynamics within households and influence how child abuse cases are perceived and reported [22]. Oman and many nearby Arab countries have enacted comprehensive child protection laws that define child abuse, outline reporting mechanisms, and establish legal consequences for perpetrators [23]. Government agencies and departments oversee the welfare and safety of children, ensure their well-being, and investigate reports of maltreatment [24].

Oman has ratified the CRC treaty [23], and this was followed by the establishment of a medical-legal clause for sexual assault [24]. According to this clause, there are three forms of child abuse under the Oman penal code. These are, as stated in Article 7 of the Oman Children's Law [25], violence (physical force or violence inflicted on the child), exploitation (exposure of the child to sexual activities, prostitution, pornography, attempted or actual sexual intercourse, and organ removal), and abuse (physical, emotional, and sexual abuse – direct or indirect – and neglect that leads to social, emotional and/or physical difficulties). According to UNICEF [26], “… Oman is one of the leading Arab countries in child protection and one of the leading countries committed to providing children with education, health care, and adequate protection”. Oman also appears to have adhered to and aspired to global initiatives aimed at addressing important global challenges, including the Millennium Development Goals (MDGs) and the Sustainable Development Goals (SDGs) [27,28]. For example, Goal 16 of the SDG emphasizes child protection and access to justice.

Little attention has been paid to understanding the characteristics of CSA victims and perpetrators, as well as the specific medical and judicial outcomes for the Omani population. Some experts who study Arab and Islamic cultures have suggested that the social and cultural norms prevalent in these regions may hinder the effective enforcement of existing laws designed to protect victims of CSA [15,24,29]. This, in turn, can lead to a significant gap between the laws in the books and the way CSA cases are handled by law enforcement, prosecution, and courts [30].

Data on CSA worldwide have indicated a complex gender dynamic. Although both boys and girls are vulnerable to CSA, studies show variations in prevalence rates and reporting behaviors. Most existing data on gender and CSA suggest that girls are more commonly identified as victims of CSA than boys [31]. This trend may reflect differences in reporting behaviors rather than actual occurrence rates, influenced by sociocultural factors. Gender differences in reported cases of CSA could arise from differences in both the prevalence of CSA and the reporting rates. Given that men are more likely to be perpetrators than women and that heterosexual orientation is more common than homosexual ones, it is logical that studies often report more girls than boys as victims. However, this does not preclude the possibility of under-reporting cases involving boys due to factors such as homophobia and stereotypes about who can be a victim of sexual violence. In the Omani context, traditional gender expectations and social norms could influence both the perception and the reporting of CSA. For boys, stigma related to masculinity and social norms could lead to under-reporting, while for girls, concerns about chastity, marriage prospects, and virginity could similarly inhibit reporting. Additionally, the observed trend of more cases of CSA occurring among girls could be influenced by gender segregation, which affects the frequency of contact between boys and strangers. In Oman, where gender segregation is prevalent, girls may have fewer interactions with strangers, which could impact the incidence of reported CSA. On the contrary, men may engage in more homosexual contact due to gender segregation, which could also affect the person.

Currently, there is limited information on the gender dynamics of reporting behavior and medical-legal outcomes in countries of the Arabian Gulf. Given these gaps, more research is warranted on these issues in the Omani context to better understand the factors that influence the reporting and incidence of CSA.

Within the background mentioned above, this study aims to provide a comprehensive description of the characteristics of victims and perpetrators of CSA in the context of Oman. This includes factors such as age, sex, and the types of CSA cases encountered. The second objective aims to investigate the factors that influence the medicolegal findings and judicial outcomes of CSA complainants in Oman. This involves exploring various variables that could affect the likelihood of a favorable outcome for victims in legal proceedings.

## 2 Methodology

### 2.1 Study design, setting and duration

This is a retrospective study of CSA cases that were reported in Oman over a year between January 2017 and December 2017. The cases in the present study were derived from records from Oman's Prosecutorial Office, which were accessed by one of the authors (MM), who was a practicing prosecutor in the Oman judicial authority. Before designing the collection of information from official records, the authors, some of whom are members of the National Forensic Committee and considered experts in child sexual abuse, met twice to deliberate on the content of the study. Finally, 269 cases of CSA that comprised the subject of this study were extracted, along with relevant information.

Oman has centralized but compartmentalized government services and agencies. In the 11 governorates, Child Protection Committees (CPC) are established to prevent and address child abuse. The 2014 Child Law, along with its 2019 executive regulations, prohibits any form of violence against children, including in schools. According to this law, reporting any incident of child abuse is mandatory and allows children to be removed from violent situations.

In the community, a significant figure, for that matter, the parent or sibling of the abused child, is required to report the incident to the police or take the abused child to the nearest health facility. Oman has a universal free healthcare system. Alternatively, the ‘whistleblower’ can use the existing hotline to directly inform the Child Protection Unit under the auspices of the Ministry of Social Development.

If the abused child is identified at school, then teachers must contact the regional CPC or the nearest healthcare facility. If the abused child is identified in a healthcare facility, then doctors are required to document a physical examination. The Ministry of Health has created a manual titled "Clinical Guidelines on Child Abuse and Neglect." The manual includes a step-by-step procedure for physical examination in cases of sexual violence [32]. Like their counterparts in educational settings, healthcare workers are required to inform the Regional Hospital Task Force on Child Abuse and Child Protection Committee.

Governmental authorities in various sectors, such as health, education, social and judicial, regularly participate in refresher courses to stay up-to-date on different aspects of evidence-based child abuse and neglect. If the CSA is confirmed by cross-sectoral authorities, the suspected case is then forwarded to the criminal justice system, which, in turn, works in collaboration with the National Forensic Committee to further scrutinize the allegation. For the sake of conciseness, the National Forensic Committee has dual mandates on behalf of victims of child abuse. One is to review the physical evidence accrued from medical services or other relevant bodies that have collected evidence of child abuse. If necessary, the child is considered conducive to a forensic interview. The primary aim of a forensic interview is to gather accurate and reliable information from the child in a supportive and non-leading manner. According to the ***United States Children's Bureau*** [33], forensic interviews are crucial to the investigative and legal process when child sexual abuse is suspected. These interviews are conducted by child welfare professionals to obtain a detailed and accurate account of the child's experience of sexual abuse or exploitation.

According to the Manual entitled ***Clinical Guidelines on Child Abuse and Neglect*** published by the Ministry of Health [32], this can include signs of sexual assault, physical trauma, bruises, or other injuries that are consistent with the child's statement of abuse. As has been previously documented, an exam can be significant in a legal case, as it provides corroborating physical evidence that supports the child's allegations and can be used in court to strengthen the case against the alleged perpetrator.

### 2.2 Inclusion/exclusion criteria

In this study, participants were selected based on specific inclusion and exclusion criteria to ensure a relevant and focused sample. To be eligible, individuals had to be under 18 years old and identify themselves as Omani. Participants must be able to disclose their experience of sexual abuse or have formal confirmation of abuse through official reports, legal proceedings, or clinical evaluations. The excluded criteria included people 18 years or older, those who did not disclose their abuse experience, and those without formal confirmation of abuse. Furthermore, individuals who did not meet any of the specified criteria were excluded from the study. These criteria were established to create a well-defined participant group, impacting the composition of the cases and ensuring that the study findings are relevant and accurate about the experiences of the targeted population.

### 2.3 Demographic and clinical variables

This study included all cases of victims under 18 years of age whose records were registered with the Public Prosecution Office from January to December 2017. From these records, the sociodemographic characteristics of both the victims and defendants (or, alternatively, the perpetrators labeled) of CSA were determined. Information was extracted that had a direct bearing on medical legal findings and judicial results. The detailed contents of the study are explained in the following and are shown in Table 1. Sociodemographic variables that were extracted from the record at the Public Prosecutor's Office included: gender and age of the accused assailant and assaulted (male/female), place of occurrence (urban/rural), relationship with the offenders (intra-familial or extra-familial), and types of abuse (sodomization, vaginal coitus). For cases where there was no substantial physical evidence of either form of penetration, in the present investigation, these types of sexual abuse were labeled inappropriate sexual behavior towards children, including unwanted sexual behaviours and comments from others that make one uncomfortable or fearful [34].

**Table 1:** Sociodemographic and clinical characteristics of victims and perpetrators (n = 269).

Characteristics	Category	Number	Percentage
Locale	Urban	153	56.9
	Rural	116	43.1
The sex of the perpetrator	Male	269	100.0
	Female	0	0.0
Victim sex	Male	150	55.8
	Female	119	44.2
Age of the victim (years)	3–7	43	16.0
	8–12	87	32.3
	13–17	139	51.7
Mean (SD), Range	12.0 (3.9)		
Age of the perpetrator (years)	12–19	55	20.4
	20–29	121	45.0
	30–39	58	21.6
	40+	35	13.0
Age mean (SD), range	28.3 (10.5)		
Relationship with the perpetrator	Intrafamilial	9	3.3
	Extra-familial	260	96.7
Types of abuse	Sodomization	70	26.0
	Vaginal coitus	19	7.1
	Inappropriate sexual behaviors	180	66.9
Public prosecution	Reserved	93	34.6
	Sent to the court	176	65.4
Legal outcome (n = 176)	Innocent	42	23.9
	Convicted	134	76.1
Jail duration (years) (n = 134)	<2 years	29	21.6
	2–5 years	91	67.9
	6–10 years	4	3.0
	Unknown	10	7.5

This study explored the role of the rural and urban dichotomy in factors that lead to sexual assault and the forensic results of the courts. Most of the population of Oman resides in the northern part of the country, the Gulf of Oman, overlooking the Arabian Sea. The region is intruded by the Hajjar Mountain from the empty quarter. Unlike other emerging economies where urbanisation surges around the nation's capital or commercial cities, Oman appears to have horizontal development in both rural and urban areas. Oman has 11 administrative regions: Muscat, Musandam, Buraymi, South Batinah, North Batinah, South Sharqiyah, North Sharqiyah, Dakhiliyah, Dhahirah, Wusta, and Dhofar.

The outcome and disposition of the medical and judicial settings were explored as follows: Send for public prosecution (reserved/sent to court); Legal outcome (innocent/condemned). The duration of the jail term, as well as the reduced terms, was also used and analyzed. A court of the judicial system, the term reserved implies that the decision is not given immediately after a case is argued, but is kept under consideration by a judge or panel until a later date when a written decision is provided. Therefore, by default, these reversed cases occurred beyond the present study period, that is, between January 2017 and December 2017.

### 2.4 Statistical analysis

Descriptive statistics (e.g., frequency, percentage, mean and standard deviation (SD)) were used to explore victims' and perpetrator profiles according to selected sociodemographic characteristics. To examine the variations in the proportion of positive or negative results of the forensic examination and in legal outcomes (convicted or not convicted), a bivariate analysis of the legal outcomes of CSA cases was performed on the sociodemographic characteristics of victims and perpetrators. Statistically significant variations of the legal outcome results between sociodemographic characteristics were tested using the Chi-square (χ^2^)/Fisher's exact χ^2^ test as all variables considered in this study were categorical. A p-value <0.05 was considered statistically significant. Furthermore, multiple logistic regression analysis was performed to identify significant predictors of conviction after controlling for the effects of background characteristics. IBM SPSS 23.0 (IBM SPSS Inc., Chicago, IL, USA) was used to perform all data analysis.

### 2.5 Ethical approval

Ethical approval was granted by the Ethics and Research Committee of the Ministry of Health (MoH/DGPS/CSR/18/7063), and official permission was obtained from the General Public Prosecutor. The study was carried out in accordance with the Declaration of Helsinki and the American Psychological Association on ethical human research, including confidentiality, privacy, and data management.

## 3 Result

As shown in Table 1, the study consisted of 269 cases, with ages ranging from 3 to 17 years. Slightly more than half (52 %) of the victims were children aged 13–17 years and almost a third (32.3 %) were 8–12 years old. The mean age of the victims was 12 (±3.9) years. More than half (56 %) of the victims were male, while all the assailants were male. Most (76.6 %) of the assailants were between 20 and 40 years old. The mean age of the assailants was 28.3 (±10.5) years. Most of the cases were reported in urban areas (57 %; n = 153). Most cases of CSA were extra-familial (96.7 %) rather than intrafamilial. The types of penetrative sexual abuse included sodomization (26 %; n = 70) and vaginal coitus (7.1 %; n = 19), while the most common type of abuse, in general, was inappropriate sexual behavior (67 %; n = 180).

Of the people who were sent to court, three-quarters (76 %, 134/176) of them were convicted with different jail terms. Most (68 %, 91/134) of the convicted assailants were jailed for 2–5 years. The median jail term for those convicted was 30 months (IQR = 36), while 17 % (10/134) of the convicted assailants received reduced jail terms.

The results in Table 2 revealed that the conviction rate was higher among cases of CSA related to urban areas, male victims, older victims, younger assailants and sodomization. However, multiple logistic regression analysis identified the age of the victims, the types of the abuse, and the results of forensic tests as significant predictors of conviction. Cases involving victims aged 13–17 years had approximately 3.5 times higher odds of conviction compared to CSA cases involving victims aged 3–7 years (AOR = 3.45, 95 % CI = 1.22–9.79).

**Table 2:** Percentage of convicted and non-convicted cases by background characteristics of victims and perpetrators, and results of the binary logistic regression analysis showing the odds of convicted cases with a 95 % confidence interval (CI) (n = 176).

Characteristics	Legal outcomes					Adjusted Odds Ratio of Conviction with 95 % CI		
	Not convicted	Convicted	Total	Number	p-value	AOR	95%CI	P-value
Total	23.9	76.1	100.0	176				
**Locale**					0.171			
Urban	20.4	79.6	100.0	108		1.35	0.50–3.64	0.554
Rural	29.4	70.6	100.0	68		1.00		
**Victim sex**					0.437			
Male	21.5	78.5	100.0	93		2.77	0.98–7.90	0.560
Female	26.5	73.5	100.0	83		1.00		
Age of the victim					0.004			
3–7 (ref.)	46.2	53.8	100.0	26		1.00		
8-12	27.3	72.7	100.0	55		1.83	0.66–5.06	0.118
13-17	15.8	84.2	100.0	95		3.46	1.22–9.79	0.006
Age of perpetrator					0.291			
12-19	20.0	80.0	100.0	40		1.48	0.52–4.25	0.464
20-29	19.5	80.5	100.0	77		0.34	0.08–1.39	0.134
30-39	29.7	70.3	100.0	37		1.46	0.29–7.21	0.643
40+ (ref.)	36.4	63.6	100.0	22		1.00		
Relationships					0.674			
Intrafamilial	16.7	83.3	100.0	6		1.47	0.13–6.57	0.755
Extra-familial	24.1	75.9	100.0	170		1.00		
Type of abuse					0.072			
Sodomization	14.8	85.2	100.0	61		1.95	1.08–6.78	0.014
Vaginal coitus	18.8	81.3	100.0	16		1.34	0.43–5.82	0.390
Inappropriate sexual behaviours (ref.)	30.3	69.7	100.0	99		1.00		

Sodomization cases were 1.95 times more likely to lead to conviction than cases (AOR = 1.95, 95%CI = 1.08–6.78), such as cases of inappropriate sexual behavior or vaginal coitus (Table 2).

## 4 Discussion

When gleaned through the demographic transition model, Oman and its neighboring countries in the Arabian Peninsula are going through stage 2 of the demographic transition characterized by high birth rates, declining death rates, and ‘youth bulge’ with a pyramidal population structure, and these regions have been called 'home to one of the youngest populations in the world’ [35,36]. This demographic trend has coincided with something similar to ‘perestroika’ in the discourse relevant to recognition and identification, as well as considering a mechanism to prevent and mitigate various aspects of child maltreatment [23,[37], [38], [39]]. Al Eissa et al. [4] have explored child maltreatment prevention programs (CMP) in countries of the Arabian Gulf, including Oman. This study reported that the region falls within the moderate to fair readiness level for the implementation of evidence-based CMP. Despite the aforementioned perestroika, the discourse on child sexual abuse (CSA) remains muted, and comprehensive studies are lacking in the region, in particular.

Some studies have emerged to decipher why there is a conspicuous absence of enlightened discourse on CSA. Elghossain et al. [40] have conducted a systematic review of the prevalence of violence against adolescents in 22 countries of the Arab League. The collected data appear to suggest that maltreatment appears to be widespread in the region of interest, but there is a conspicuous absence of reporting sexual abuse. In support of this view, the lower reported rate of sexual abuse has also been documented in a study by Almazeedi et al. [41] among first-year students in higher education in Kuwait. Rather than simply indicating that it is lower in the Arabic-speaking population, some authors have begun to understand the reason behind the reported lower rate of child sexual abuse. Attrash-Najjar & Katz [42] have reported a systematic review of the literature to explore child sexual abuse in Arab societies, and 57 studies were deemed adequate for qualitative analysis. The authors identified two key obstacles to the examination of child sexual abuse in studies originating in the Arab population. The first barrier revolves around the participants' inclination to be discrete when discussing child sexual abuse. In contrast, researchers are often reluctant to discuss these sensitive topics with study participants due to social taboos. The hidden nature of child sexual abuse compounds its prevalence assessment due to cultural norms, social stigma, and lack of awareness that contribute to underreporting. Concerns about social reputation and family honor have been speculated to tend to discourage the reporting of CSA [43]. Consequently, numerous cases may go unnoticed and, therefore, the health educator may not find a reason to increase awareness. However, in their 2022 study, Brahim et al. [44] conducted a retrospective survey covering eight years from 2009 to 2016. Their research focused on women who suffered sexual assaults and sought help in the Department of Forensic Medicine in Kairouan, Tunisia. The study revealed the prevalence of various forms of physical sexual assault among these victims. This suggests that instances of sexual abuse with conspicuous signs that require medical or forensic attention were more likely to be documented and explain the magnitude of sexual abuse. In this context, it is not unusual to gather the extent of child abuse in some societies. Therefore, it appears that the only way to gauge the extent of child sexual abuse is through the reported case, as in the present study, with all the limitations that this could entail. Therefore, this study aimed to describe the characteristics of the assailant and assault in registered cases of child sexual abuse. Factors associated with the outcome of associated medical legal examinations and judicial trials were also examined.

This study analyzed 269 cases of CSA and their registered assailants. In other populations, sexual predators can be of any sex, but are predominantly male [45], since less than 4 % of CSA are perpetrated by female offenders [46]. In the present study, the assailants were males. It is not clear the reason behind it, but speculation abounds and it is worthwhile to highlight them here. Traditional gender roles and stereotypes can also play a role in shaping reporting patterns. Therefore, cultural norms or social expectations likely discourage victims from speaking out, especially when the perpetrator is female. There may be a perception that men are more likely to have a risk of child abuse, which can influence reporting patterns and law enforcement priorities [47]. In certain situations, men may have greater access or opportunity to engage in child abuse, such as in positions of authority or trust [48]. Therefore, future studies are warranted to determine whether there is an interaction between culture and gender of the offender, as the present cohort did not have female assailants.

On the victim's side, the reported cases presented here indicate that the majority were male (55.8 %). In the literature, studies show that girls are at least twice as likely to be sexually abused as boys, but the prevalence of sexual abuse among girls is higher than among boys, regardless of the definition of CSA, cultural factors, legal regulations, or the method used to estimate prevalence and incidence rates [49].

The preponderance of boys in reported cases of child sexual abuse in Arab/Islamic populated areas can be attributed to a combination of cultural, social and legal factors. First, traditional gender roles and expectations in Arab/Islamic societies often place greater emphasis on protecting and preserving the honour and reputation of girls and women. Therefore, the family can underreport the occurrence of sexual abuse by women, which, in turn, is likely to be higher among boys.

The victims were also more likely to be 13–17 years of age or 8–12 years of age. In existing statistics and literature, while all children under 18 years of age are at risk of victimization, children between 6 and 12 years of age are at the highest risk of first encounter with CSA [50].

In the present study, a seemingly contradictory finding is that 96.7 % of the reported cases are those of extra-familial abuse. This contrasts with the existing literature, where there is a higher prevalence of intrafamily sexual abuse compared to the results of the present study [51]. It is not apparent why this could be the case, but some speculations are worth mentioning. In general, it is widely believed that intrafamilial CSA are underreported due to relatively elevated feelings of fear, shame, ostracism, and family loyalty of victims compared to extrafamilial CSA [52]. Intrafamilial perpetrators also frequently use 'not to tell' instructions, as well as cause greater levels of physical and emotional injury to their victims compared to extrafamilial abuse [53]. Furthermore, it is possible that in a traditional society such as Oman, where extended family, tribal identity, and kinship are widely present, a clear distinction between intrafamilial and extra-familial sexual abuse is difficult to apply. Being a collective society with a strong tendency for individual identities to be intertwined with those of the family [54], incest and sexual abuse considered incest tend to be more stigmatized [55]. Therefore, it is possible that when sexual abuse is reported and documented, the family is likely to discount their kinship with the assailant or simply put, such cases are likely to be underreported.

In a review of Arab culture by Polonko et al. [56], the patriarchal nature of these societies that leads to female segregation and decreased exposure to non-family members could result in a lower prevalence of intrafamilial CSA among girls in Middle Eastern countries. In the present study, this could also explain the higher prevalence of extrafamilial CSA, with the highest reported cases among male victims [55.8 %]. Existing prevalence data from different countries indicate that CSA gender differences vary between countries and are significantly impacted by regional cultural and social values [57]. Another factor that results in the higher reported prevalence of male victims may be the tendency of traditional Arab societies to respect the sexual honor of women as a reflection of their entire family, which could also lead to under-reporting [58]. Although the findings of the present study on CSA cases brought to medical and legal attention indicate that there are slightly more male victims of CSA [56 %], a sentinel study of victims who were referred to health centers in Oman indicates a higher number of female victims of CSA (64 %) [23]. This may indicate that female victims are less likely to publicly accuse their assailants and to undergo legal proceedings. The possible reasons for this need to be further explored among similar demographic populations in studies better equipped to examine this finding.

A 15-year comparative study [59] in five Western countries showed that only 14 % of sexual violence cases are reported, of which only 30 % proceed to prosecution, 20 % undergo court adjudication, and 12.5 % are convicted of any sexual offence charged. In terms of CSA, studies indicate that fewer than half of sexual assaults reported to the police result in prosecution or charges and less than a third of these cases result in convictions, with evidence of physical injury highly associated with positive legal outcomes [7]. In the present cohort, approximately 65.4 % of the cases were tried in court. Among these, 76.1 % (n = 134) were convicted and sentenced to an average jail term of 30 months, ranging from 2 to 5 years. In an Omani law penal code, any person who has committed acts relevant to sexual intercourse without consent, or 'Offenses against Honor,’ will be punished in prison for a period not less than ten years and not more than 15 years, with the additional clause that consent is not recognized if the victim is under 18 years of age. Furthermore, if the victim is under 15 years of age or if the perpetrator is the victim's legal guardian, then they will be punished with life imprisonment [25,60]. In the present survey, perpetrators, on average, were sentenced to jail terms less than those stipulated in the penal code. Furthermore, 17 % of those who were convicted eventually reduced their jail term.

According to the Oman judiciary, adolescents under 18 years of age are considered minors, but anyone over 9 years of age is liable for criminal responsibility [61]. This is just a slightly older age compared to the perpetrators of CSA from other countries, such as the mean perpetrator age of 23.8 years reported in a sample from the United States [62], or the mean age of 25.2 years in a sample from Egypt [63]. Marshall and Barbaree's 1990 integrated theory of sexual offenses suggests that CSA perpetrators who are victims of childhood maltreatment themselves are at increased risk of developing poor social control and distorted emotional self-regulation during adolescence, ultimately leading to the development of false fantasies and sexual offenses after the transition to early adulthood [64].

The final interrelated objective of the present study is to determine whether there were specific factors that ultimately led to prosecution and conviction. First, having positive forensic outcomes appears to have a direct impact on conviction. This result echoes the conclusion of Palusci et al. [65] that cases with positive medical legal outcomes were 2.5 times more likely to be prosecuted and convicted. Second, victims aged 13–17 years were more likely to have their perpetrators convicted. A third factor is the type of CSA, and this study suggests that sodomization cases were significantly associated with a conviction, more than vaginal coitus or inappropriate sexual behavior. In contrast to this, a study reported by Saint-Martin et al. [16] on sexual assailants in France reported that types of abuse do not have a singlehanded overbearing on conviction. Jong et al. [66] have suggested that the quality of verbal evidence and the effective testimony of child victims ensure that perpetrators are successfully prosecuted. Sodomy is prohibited and is perceived as repulsive in sociocultural teachings in Islamic societies [67]. It is not clear whether such sociocultural factors influence which types of CSA are more likely to lead to prosecution. Given that this is a preliminary study, there is a pressing need for more research to develop a comprehensive database on the patterns and outcomes of child sexual abuse in Oman.

### 4.1 Limitations

One limitation of this study is its relatively narrow time frame, covering one year from January 2017 to December 2017. The study acknowledges that 93 cases were reserved due to this limited time frame. This limitation implies that the findings may not fully capture the dynamics and variations in CSA cases over a longer period. Reserved cases might have additional insights and nuances that could contribute to a more comprehensive understanding of the issue. To enhance the robustness and generalizability of future research, it is recommended that subsequent studies extend their data collection over longer periods. In relation to this, this study is limited by the fact that this study relies on data extracted from the prosecution's office records. The use of retrospective data introduces the possibility of selection bias. Therefore, only reported cases are included in the study, which could omit unreported cases or cases that were not prosecuted. This can limit the generalizability of the findings to the entire population of CSA cases in Oman. The study relied on available data, but unfortunately, the prosecution's office records did not include specific details on where and when child sexual abuse incidents occurred. This limited our ability to conduct a more comprehensive analysis of these aspects, which could have helped us better understand the dynamics of CSA in Oman. To gain a deeper understanding of the context and patterns of CSA cases, future research should prioritise collecting more detailed data. Linked to these, the present data is based on quantitative data and do not include qualitative insights from victims, perpetrators, or other stakeholders. Future studies would also use qualitative data to better understand the context and motivations behind the CSA cases. Second, the accuracy and completeness of the data in the prosecution's office records may be a limitation. Data entry errors, missing information, or variations in record-keeping practises at different locations or time periods can affect the reliability of the findings. Third, the study covers a specific one-year period (January to December 2017). Although this period of time may provide insight into trends during that period, it may not capture long-term or evolving patterns in child sexual abuse. The social, legal and cultural factors that influence the cases of CSA can change over time. The study's findings may not reflect more recent developments or changes in reporting and prosecution practises. The study includes 269 cases of CSA. The sample size may limit the ability to detect statistically significant differences in subgroups or rare outcomes, and the findings may not be representative of the entire population. Fourth, the binary logistic regression currently employed assumes linearity and independence in the data set, and violations of these assumptions can lead to issues such as overfitting and multicollinearity. Additionally, binary logistic regression is used primarily to establish associations, but cannot establish causality due to its correlational nature. Lastly, the present discourse assumes that all suspected cases of CSA are genuine and thus the lack of prosecution is due to a lack of evidence or a misinterpretation. The corollary is that unfounded allegations may not necessarily imply that CSA did not occur. The question of how to disentangle this issue remains enigmatic. There are likely to be many factors that could contribute to persecution or otherwise, including misinterpretations, false claims for personal gain, lying older children, or simply having a smart lawyer. Therefore, relying solely on CSA evidence to determine the inclusion/exclusion of the case is likely to be marred by problems. Thus, the selection criteria used may result in the exclusion/inclusion of certain cases, potentially skewing the sample. In future studies, an in-depth exploration of these issues is warranted.

## 5 Conclusion

To date, this is the first study to specifically examine the forensic and judicial outcomes of perpetrators of child sexual abuse. Oman and its neighboring Arab countries have recently introduced various best practises to protect the well-being of its children, including those who have been subjected to sexual abuse. Thus, the law establishes legal recourse for such offenses. Overall, this sentinel study appears to suggest that Oman has a functional system to punish those who commit child sexual abuse, although it may be perceived as rudimental. Some of the present findings appear to be culturally specific, while other aspects appear to echo international trends. Being a preliminary study, more studies are therefore warranted to establish a database on the trajectories of child sexual abuse in this region.

## CRediT authorship contribution statement

**Muna Alshekaili:** Writing – original draft, Methodology, Investigation, Formal analysis, Data curation, Conceptualization. **Mohammed Ali Al-Marzoqi:** Project administration, Methodology, Data curation, Conceptualization. **Salim Al-Huseini:** Project administration, Methodology, Investigation, Data curation. **M Mazharul Islam:** Project administration, Methodology, Investigation, Formal analysis. **Fatima Al-Sulaimani:** Formal analysis, Data curation. **Walid Hassan:** Resources, Formal analysis, Data curation, Conceptualization. **Yahya Alkalbani:** Methodology, Investigation. **Mohamed Al Breiki:** Project administration, Investigation. **Abdullah Al-Madhani:** Project administration, Methodology, Investigation. **Nithila Mariam Roy:** Writing – original draft, Methodology. **Ibrahim Al-Zakwani:** Writing – original draft, Methodology, Formal analysis. **Aishwarya Ganesh:** Writing – review & editing, Methodology. **Samir Al-Adawi:** Writing – review & editing, Writing – original draft, Methodology, Formal analysis.

## Data availability

All data generated or analyzed during this study are included in this article. Further information could be made available on request with the corresponding author.

## Funding

None.

## Declaration of competing interest

The authors declare that they have no known competing financial interests or personal relationships that could have appeared to influence the work reported in this paper.
